# Effective Antimicrobial Activity of Plectasin-Derived Antimicrobial Peptides against *Staphylococcus aureus* Infection in Mammary Glands

**DOI:** 10.3389/fmicb.2017.02386

**Published:** 2017-12-04

**Authors:** Lianbin Li, Liangliang Wang, Yuqi Gao, Jianhua Wang, Xin Zhao

**Affiliations:** ^1^College of Animal Science and Technology, Northwest A&F University, Xianyang, China; ^2^School of Pharmaceutical Sciences, Tsinghua University, Beijing, China; ^3^Gene Engineering Laboratory, Feed Research Institute, Chinese Academy of Agricultural Sciences, Beijing, China; ^4^Department of Animal Science, McGill University, Montreal, QC, Canada

**Keywords:** *Staphylococcus aureus*, NZ2114, MP1102, antimicrobial peptides, mastitis, mammary glands

## Abstract

*Staphylococcus aureus* (*S. aureus*) is the causative agent for a wide variety of illnesses ranging from minor skin infections to life-threatening diseases. Development of antibiotic resistance by the bacteria has rendered many antibiotics ineffective. It has been known that plectasin-derived antimicrobial peptides (AMPs; NZ2114 and MP1102) are promising alternatives to antibiotics. However, their activities against *S. aureus* in mammary glands were unknown. Our objective was to assess the antimicrobial activities of NZ2114 and MP1102 against *S. aureus* in milk, in cultured mammary epithelial cells, and in a mouse model in order to evaluate their potentials as anti-mastitis agents. NZ2114 and MP1102 showed *in vitro* bactericidal effects against *S. aureus* in both the culture medium and the milk. NZ2114 and MP1102 at the concentration of 100 μg/mL reduced the number of *S. aureus* by almost 100% within 4 h in processed bovine milk. Similarly, both NZ2114 and MP1102 were efficient to reduce the number of internalized *S. aureus* in cultured mammary epithelial cells. Finally, both AMPs significantly reduced the *S. aureus* load and concentrations of TNF-α and IL-6 in mammary glands, compared to a buffer control in the mouse model. Our results suggest that NZ2114 and MP1102 may be used to treat *S. aureus*-induced mastitis.

## Introduction

*Staphylococcus aureus* (*S. aureus*) causes a wide-spectrum of infections in both humans and domesticated animals. Although *S. aureus* is generally regarded as an extracellular pathogen, the ability of *S. aureus* to invade and thrive intracellularly plays a critical role in the persistent and recurrent cases of infections (Clement et al., 2005). The best known *S. aureus-*induced intracellular infection is mastitis in dairy cattle. The pathogenicity and involvement of *S. aureus* as a major causative pathogen of chronic subclinical mastitis has been widely investigated. However, the treatment using antibiotics is usually ineffective.

Antimicrobial peptides (AMPs) are a new class of antimicrobial agents with a new mode of action and appear as one of the most promising antimicrobial medicines (Hancock and Sahl, 2006). Plectasin with 40 amino acids is a cationic AMP isolated from the saprophytic ascomycete *Pseudoplectania nigrella* and has a potent activity against gram-positive bacteria (Mygind et al., 2005). Most AMPs have been thought to target the bacterial cell membrane. Interestingly, plectasin can interfere with cell wall synthesis by specifically binding to Lipid II which is the key of bacterial cell wall precursor (Schneider et al., 2010). Despite its amphipathic nature, plectasin does not compromise membrane integrity, thus reducing the risk of unspecific toxicity. NZ2114 is a variant of plectasin (Andes et al., 2009), with three mutational sites. The susceptibility testing has shown that NZ2114 possesses a stronger activity against Staphylococci than plectasin (Zhang et al., 2014). Moreover, earlier reports showed that NZ2114 could effectively target intracellular *S. aureus* in the human THP-1 monocytes (Brinch et al., 2010). MP1102 was further modified from NZ2114 through increasing its α-helicity index and hydrophobic moment and consequently it exhibited a stronger activity against 20 clinical isolates of methicillin-resistant *S. aureus* than NZ2114 (Zhang et al., 2015).

The various ingredients of the milk may impair the efficacy of any anti-mastitis agents, thus having a high activity in milk is a basic requirement for anti-mastitis drugs administered through the mammary gland (Schmelcher et al., 2015). Similarly, an anti-staphylococcal agent should also be screened for its intracellular activity, considering the fact that *S. aureus* can invade and survive inside cells such as mammary epithelial cells. Nevertheless, *ex vivo* experiments in cow milk and mammary epithelial cells cannot completely mimic the complex situation within a cow’s udder. However, screening antimicrobial agents using intramammary infected cows is associated with high costs and complex management issues. The mouse model of infectious mastitis can be used as a prelude to the study of bovine mastitis. Numerous studies have adopted this strategy for exploring the effect of various antibiotics (Demon et al., 2012; Fu et al., 2014; Nazemi et al., 2014).

To the best of our knowledge, there are few studies to date reporting the use of AMPs in the treatment of mammary gland infection. This study was the first to evaluate the potential of two plectasin-derived antimicrobials peptides, NZ2114 and MP1102, as antimicrobial drugs against *S. aureus*-induced mastitis using both *in vitro* studies and a mouse model.

## Materials and Methods

### Bacterial Strains, Sources of Antimicrobial Agents, and Antimicrobial Assays

*Staphylococcus aureus* E48, a clinical mastitis isolate, had been used to induce mouse mastitis model (Wang et al., 2017). Tetracycline is one of the most extensively used antibiotics for mastitis treatment because of its relative safety, low cost, and a broad-spectrum activity (Kuang et al., 2009). It was used as a positive control in this study and was purchased from Sigma–Aldrich. The AMPs were prepared according to a previously described protocol (Zhang et al., 2015). The minimum inhibitory concentrations (MICs) were used to evaluate the antibacterial activities of antimicrobial agents (Wiegand et al., 2008). Briefly, *S. aureus* were grown to the log-phase at 37°C in the Mueller–Hinton broth, and diluted to 5 × 10^5^ CFU/mL with the fresh medium. A total of 180 μL of cell suspension and 20 μL of serially twofold diluted peptide were added to each well, and the plates were incubated at 37°C for 16–20 h. The final concentration of antimicrobial agents was prepared with gradients of 64, 32, 16, 8, 4, 2, 1, 0.5, 0.25, 0.125, and 0.0625 μg/mL. Three replicates were examined for each concentration. The MICs refer to the lowest concentration where no growth was visible after 16–20 h of incubation.

### Time-Kill Kinetics

Time-kill assays of NZ2114, MP1102, and tetracycline were performed to evaluate the *in vitro* bactericidal activities. *S. aureus* E48 at the concentration of 5 × 10^5^ CFU/mL in fresh TSB medium was added to a flask, together with antimicrobial agents at concentrations of 0.5, 1, or 5 × MIC. The mixture was cultured at 37°C with shaking for 24 h. At 0, 2, 4, 8, 12, and 24 h of incubation, 100 μL sample was taken from each flask, serially diluted in sterile phosphate-buffered saline (PBS), plated onto tryptic soy agar (TSA) plates to count colonies after incubated at 37°C for 24 h. The experiments were repeated three times.

### Bactericidal Activities of Antimicrobial Agents in Bovine Milk

The activities of antimicrobial agents on *S. aureus* in bovine milk were determined in commercial whole-fat ultra-high-temperature-sterilized (UHT) milk as described by Schmelcher et al. (2012). UHT milk (Inner Mongolia Yili Industrial Group Co., Ltd.) at 37°C was inoculated with 5 × 10^5^ CFU/mL of exponentially growing cultures of *S. aureus* E48 in the presence of antimicrobial agents (100 μg/mL) or PBS buffer (negative control). The mixture was incubated without shaking. Samples were taken at 0, 2, 3, and 4 h after inoculation, and serially diluted in sterile PBS, plated onto TSA plates to count colonies after incubated at 37°C for 24 h. The absence of *S. aureus* E48 in non-inoculated UHT milk was verified by direct plating. Three independent assays were carried out on separate days.

### Cell Culture

An established bovine mammary epithelial cell line, designated MAC-T, which has been used for *S. aureus* internalization assays (Hensen et al., 2000; Bouchard et al., 2013), was used for this experiment. The MAC-T cell was cultured in cell culture dishes in the Dulbecco’s modified eagle medium (DMEM) containing 100 U/mL penicillin, 100 μg/mL streptomycin, and 10% heat-inactivated fetal calf serum. MAC-T cells were incubated in a humidified incubator at 37°C with 5% CO_2_. After the cells were cultured to a confluent monolayer, the trypsin + EDTA solution (0.1%/0.04%) was added. The MAC-T cells were suspended in fresh medium at a concentration of 2 × 10^5^ cells/mL and cells were then seeded in 24-well plates (1 × 10^5^ cells/well) and incubated overnight at 37°C in 5% CO_2_ to obtain a confluent monolayer for the internalization assay.

The effect of peptides on the mammary epithelial cell viability was measured by using a cell counting kit-8 (CCK-8, Shanghai Bangyi Biotechnology) as described previously (Fung and Demple, 2005).

### Determination of the Intracellular Activities of the Antimicrobial Agents in Cultured Mammary Epithelial Cells

The intracellular activities of AMPs were performed as previously described (Peyrusson et al., 2015) with minor modifications. Bacteria (1 × 10^7^ CFU) were added to mammary epithelial cell cultures at a bacterium-to-epithelial cell ratio of 100:1. After 1 h, extracellular bacteria were removed by thorough washing and gentamicin. Washing was performed by rinsing a well with PBS 10 times. After removing the washing fluid, DMEM supplemented with gentamicin (100 μg/mL) was added and incubated for 2.5 h to kill the extracellular bacteria. Mammary epithelial cells with intracellular bacteria were then re-cultured in a standard culture medium. Six wells of cells were lysed immediately as described later to calculate total numbers of intracellular bacteria at 0 h. The remaining wells of cells were cultured for additional 24 h in the presence of 3 antimicrobial agents. Afterward, the cells were washed thoroughly 10 times. Then, the cells were trypsinized with 450 μL of trypsin + EDTA solution approximately 15 min at 37°C. After cells were detached, 50 μL 1% Triton X-100 at a final concentration of 0.1% (vol/vol) was added to lyse the cells during a 10-min incubation at 37°C. The number of intracellular bacteria was determined. All tests were repeated three times.

### A Mouse Model of Bovine Mastitis

The animal protocols used in this work were approved by Institutional Animal Care and Use Committee of Northwest A&F University. The 6- to 8-week-old specific-pathogen-free BALB/c mice were purchased from the Experimental Animal Center of the Medical College of Xi’an Jiao Tong University. To evaluate the efficacy of plectasin-derived AMPs in a mouse model of bovine mastitis, the pregnant female mice were randomly divided into five groups: snipped only group, *S. aureus* + PBS group, *S. aureus* + NZ2114 group, *S. aureus* + MP1102 group, and *S. aureus* + tetracycline group. The lactating mice were challenged with *S. aureus* E48 as described earlier (Schmelcher et al., 2015). The pentobarbital sodium (1.4 mg/20 g bodyweight) was used for anesthesia of the lactating mice by intraperitoneal injection. The distal end of the teat L4 (on the left) and R4 (on the right) was removed (snipped) by a small cut. These L4 and R4 glands constitute the fourth pair found from head to tail. For all groups except for the snipped only group, glands were injected with approximately 10^4^ CFU *S. aureus* in 50 μL PBS. At 45 min after *S. aureus* inoculation, the challenged glands were injected with 100 μg AMPs in 20 μL or 20 μL of PBS, depending on the treatment. The mice were euthanized 24 h after the *S. aureus* inoculation, and mammary glands were aseptically collected and weighed. Five mice (10 mammary glands) were used for each group. Seven mammary glands from each treatment were sampled and homogenized in PBS (100 mg/200 μL). Serial dilution plating on Baird-Parker agar plates was done to determine intramammary *S. aureus*. A part of the homogenate was centrifuged, and the supernatant was used for tumor necrosis factor alpha (TNF-α) and interleukin 6 (IL-6) assays, using the mouse TNF-α and IL-6 ELISA kits (Shanghai Bangyi Biotechnology). The tissue from three mammary glands from each treatment was fixed with 10% buffered formalin for histopathological analyses (HE staining).

### Statistical Analyses

The statistical procedures, means, and standard deviation (SD) were computed with SPSS 16.0. The data are expressed as mean ± SD. Comparisons between the groups were performed with ANOVA with a Duncan’s test. The differences were considered to be significant at *P <* 0.05.

## Results

### Efficacy of NZ2114 and MP1102 against *S. aureus* E48 in the Culture Medium

**FIGURE 1:**
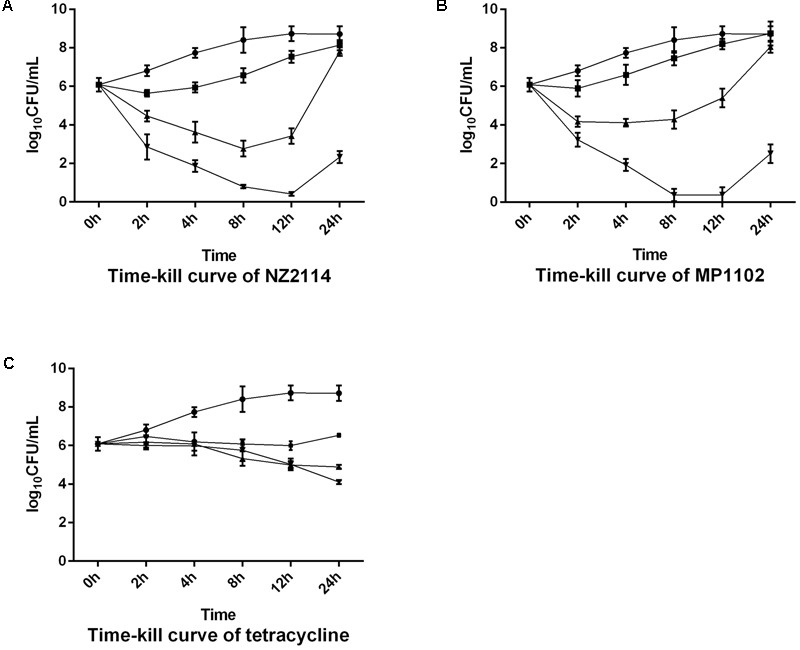
Efficacy of antimicrobials (**A**: NZ2114; **B**: MP1102; **C**: tetracycline) against *Staphylococcus aureus* E48 in the culture medium. Different concentrations were used: 0× (circles), 0.5× (squares), 1× (triangles), and 5× MIC (inverted triangles). Samples were collected at 0, 2, 4, 8, 12, and 24 h post-exposure and colony counts were determined. Each value represents the mean ± SD.

The MICs of MP1102, NZ2114, and tetracycline against *S. aureus* E48 were 1, 2, and 1 μg/mL, respectively. As shown in **Figure 1**, MP1102 and NZ2114 at the concentrations of 5×MIC most significantly decreased *S. aureus* at 12 h after inoculation. However, bacterial re-growth occurred at the 24 h time point. At 1×MIC, bacterial counts initially decreased, but increased after 8 h post-exposure. In the presence of 0.5×MIC of MP1102 or NZ2114, the *S. aureus* grew more slowly than those in the control. All concentrates of tetracycline inhibited bacterial re-growth during 24 h. The data indicate that the bactericidal activities of plectasin-derived AMPs were both dose- and time-dependent.

### Bactericidal Activities of NZ2114 and MP1102 in Bovine Milk

**FIGURE 2:**
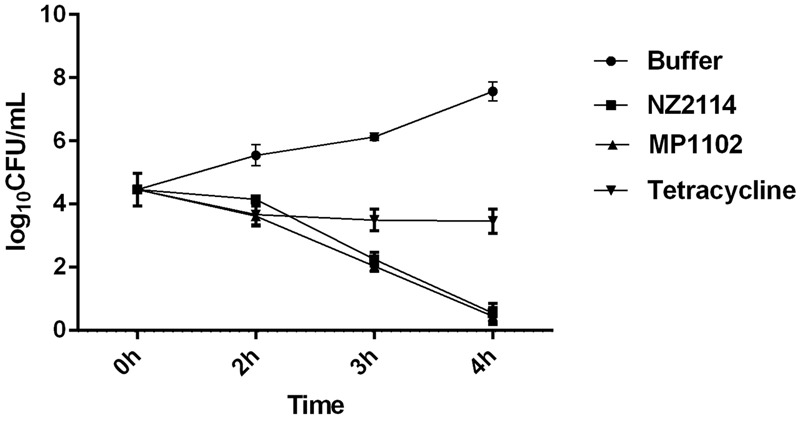
Effects of antimicrobials on concentrations of *S. aureus* E48 in bovine milk at 37°C with NZ2114 (100 μg/mL, squares), MP1102 (100 μg/mL, triangles), tetracycline (100 μg/mL, inverted triangles), or the phosphate-buffered saline (PBS) buffer (circles). Each value represents the mean ± SD.

The bactericidal activity of plectasin-derived AMPs against *S. aureus* in sterile homogenized whole milk was evaluated with the presence or absence of 100 μg/mL antimicrobials to milk at 37°C inoculated with ∼10^5^ CFU/mL of exponentially growing *S. aureus* cells. As shown in **Figure 2**, both AMPs significantly reduced bacterial numbers over the course of the experiment (4 h) as compared to the buffer control and the tetracycline group. After 4 h, there were almost no colonies recovered in the NZ2114 and MP1102 groups.

### The Bactericidal Activities of NZ2114 and MP1102 against Strain *S. aureus* E48 in Mammary Epithelial Cells

The potential cytotoxicity of NZ2114 and MP1102 was first evaluated. Cell viability was not affected by tetracycline, NZ2114, and MP1102 at the concentrations of 100 μg/mL, in comparison with the negative control without any antimicrobial components (data not shown).

**FIGURE 3:**
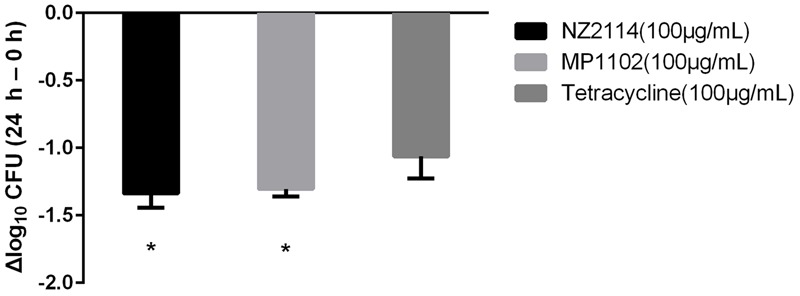
The bactericidal activities of antimicrobials against strain *S. aureus* E48 in mammary epithelial cells. The *Y*-axis shows the change in Log_10_ CFU/well after 24 h of incubation compared with the initial number of intracellular bacteria. Each value represents the mean ± SD. An asterisk (^∗^) demonstrates a significant difference (*P <* 0.05) versus the tetracycline group.

In order to determine the bactericidal activities of NZ2114 and MP1102, *S. aureus*-infected bovine mammary epithelial cells were exposed to 100 μg/mL of antimicrobials for 24 h. As shown in **Figure 3**, both NZ2114 and MP1102 were efficient to reduce the internalized *S. aureus*. NZ2114 and MP1102 were similarly efficient and better than tetracycline.

### Assessment of Antimicrobial Peptides Efficacy against *S. aureus*-Induced Mastitis in Mice

**FIGURE 4:**
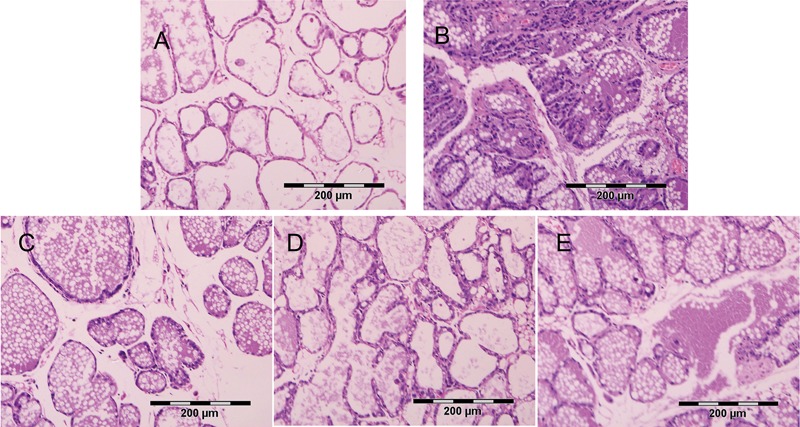
Effects of antimicrobials on mammary tissues (HE, × 100; ruler represents 200 μm). **(A)** mammary tissue of the snipped only group (no *S. aureus*), **(B)** *S. aureus* + PBS treatment, **(C)** *S. aureus* + NZ2114 treatment, **(D)** *S. aureus* + MP1102 treatment, and **(E)** *S. aureus* + tetracycline treatment.

**FIGURE 5:**
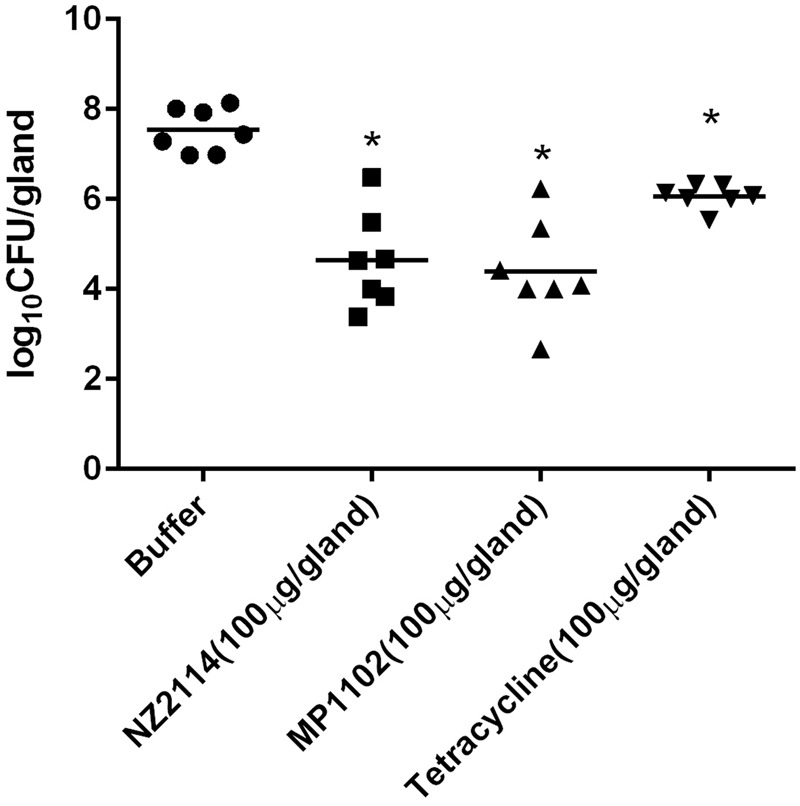
Quantification of bacterial numbers in the mammary glands in untreated *S. aureus*-infected mice (circles) and infected mice treated with NZ2114 (squares), MP1102 (triangles), or tetracycline (inverted triangles), after 24 h. An asterisk (^∗^) demonstrates a significant difference (*P <* 0.05) in comparison with the buffer group.

The *in vivo* efficacy of the AMPs against *S. aureus* in a model of mastitis was evaluated. The *S. aureus*-induced inflammatory cell infiltration was significantly reduced by both AMPs and tetracycline (**Figure 4**). The numbers of recovered *S. aureus* from the mammary tissue are shown in **Figure 5**. After 24 h, *S. aureus* numbers in the PBS control (*S. aureus* + PBS treatment) glands exceeded 3.5 × 10^7^ per mammary gland. Both peptides and tetracycline significantly (*P* < 0.05) reduced *S. aureus* numbers in the mammary glands compared to the PBS control, but the effect of tetracycline was considerably weaker than those of the AMPs (1.48, 2.9, and 3.15 log_10_ units reduction for tetracycline, NZ2114, and MP1102, respectively).

**FIGURE 6:**
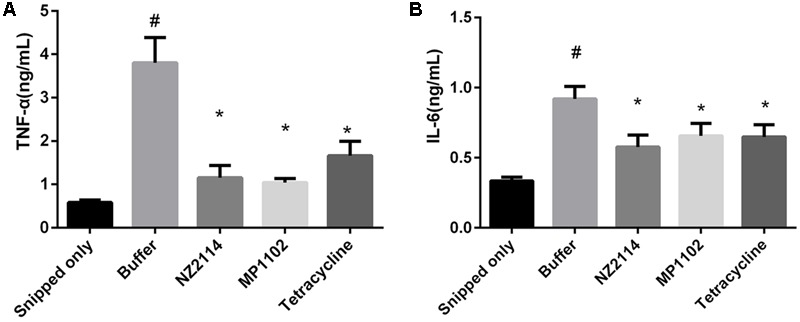
Effects of antimicrobials on intramammary **(A)** TNF-α and **(B)** IL-6 concentrations. An asterisk (^∗^) demonstrates a significant difference (*P <* 0.05) versus the buffer group. The symbol # demonstrates a significant difference (*P <* 0.05) versus the snipped only group. Each value represents the mean ± SD.

The concentrations of TNF-α and IL-6, which can be used as indicators of inflammation in mammary gland tissue, were examined (**Figure 6**). At 24 h post-infection, *S. aureus* infection without antimicrobial treatments showed a significant (*P* < 0.05) increase in TNF-α and IL-6 compared to snipped only glands with no *S. aureus* inoculation. Both AMPs and tetracycline significantly reduced TNF-α and IL-6 concentrations in comparison with the buffer control (*P* < 0.05).

## Discussion

For the dairy industry, mastitis is the most costly disease and affects animal health and welfare. Bovine mastitis is accompanied by decreased milk production, increased health care costs, higher culling rates, and sometimes even death. Moreover, bovine mastitis poses a threat to human health since it may be responsible for transfer of antimicrobial resistance and for food poisoning. *S. aureus* is the pathogen responsible for between 5 and 70% of bovine mastitis (Zecconi and Scali, 2013). *S.aureus* mastitis is difficult to be treated and prone to resurgence. The antibiotic treatments have a low cure rate with often lower than 15% in bovine *S. aureus* mastitis, which is ascribed to poor penetration of the gland by antibiotics and to the ability of *S. aureus* cells to invade phagocytic cells or mammary gland epithelial cells where they can persist for long periods (Garzoni and Kelley, 2009). AMPs appear as a promising antimicrobial agent (Narayana et al., 2015). AMPs usually do not increase bacterial mutagenesis, as they do not elicit bacterial stress pathways (Rodríguez-Rojas et al., 2014). NZ2114 and MP1102 are plectasin-derived AMPs with potent activities against gram-positive bacteria. However, no studies had evaluated the NZ2114 or MP1102 in *S. aureus* infections in mammary glands. Thus, this study represents the first to evaluate plectasin-derived AMPs for their intracellular anti-*S.aureus* activities with mammary epithelial cells.

We have shown that NZ2114 and MP1102 had intracellular bactericidal activities in bovine mammary epithelial cells. Similarly, Brinch et al. (2010) reported that NZ2114 had bactericidal activity against intracellular *S. aureus* in human THP-1 monocytes. How AMPs may enter mammalian cells is still unclear. Cell-penetrating peptides are able to penetrate the mammalian cell membrane without causing significant cytoplasmic membrane damage (Madani et al., 2011). AMPs share many structural characteristics with cell-penetrating peptides. For example, they are both short and cationic sequences with a high affinity for membranes (Henriques et al., 2006). The capacity to translocate across the mammalian cell membrane has been reported for some of these AMPs (Takeshima et al., 2003). Although the mechanism of action is not completely resolved for AMPs, our results with bovine mammary epithelial cells support the notion that both NZ2114 and MP1102 had intracellular bactericidal capacity.

This study is also the first which evaluated plectasin-derived AMPs in a mouse model of the *S. aureus*-induced mastitis. Various previous studies have used the mouse model of mastitis for investigating mammary gland infections and the efficacy of different antibiotics or lysostaphin (Brouillette et al., 2004; Schmelcher et al., 2012; Demon et al., 2013). Those studies have guided us to choose the number of bacteria used as inoculum (∼10^4^ CFU per gland) (Schmelcher et al., 2012) and the time point of antimicrobial injection (∼45 min) (Schmelcher et al., 2015). Bacterial numbers in the positive control reached approximate 10^8^ CFU/gland at 24 h post-infection and were similar to those reported previously, indicating the validity of our mouse model. NZ2114 and MP1102 were more effective than tetracycline in reducing *S. aureus* counts in mammary gland after 24 h of treatment. Different bactericidal mechanisms of the AMPs and tetracycline may be responsible for our observation. The plectasin hinders cell wall synthesis by directly binding the bacterial cell wall precursor Lipid II, whereas tetracycline affects protein translation (Schneider et al., 2010). Therefore, NZ2114 and MP1102 had a faster antimicrobial activity than tetracycline and this is supported by our time-kill results in **Figure 1**. Moreover, intracellular bactericidal activities of NZ2114 and MP1102 were lower than the extracellular bactericidal activities. This observation has been reported previously by several studies (Brinch et al., 2009, 2010).

By using both cultured mammary epithelial cells and a mouse mastitis model, our results clearly shown that plectasin-derived AMPs (NZ2114, MP1102) had intracellular bactericidal activities. Thus, they can be used to treat not only mastitis but also other intracellular *S. aureus* infections. As *S. aureus* has the ability to transiently colonize the skin and mucous membranes, it became a common cause of skin and soft tissue infections in both humans and domesticated animals. Once *S. aureus* penetrates the subcutaneous tissues and reaches the blood, it can infect almost any organ, most notably bone tissue and cardiac valves. It is responsible for the 26% of community-acquired pneumonia in the United States, and a serious threat to the survival of the patients affected by severe pneumonia (Kluytmans et al., 1997). AMPs with intracellular bactericidal activities could be used to treat these infections.

As we all know in the past, to obtain AMPs from natural sources or chemical synthesis is a time-consuming process with a high-cost. At present, recombinant DNA technology provides an economical means for protein production. Furthermore, because plectasin was of fungal origin, it could be expressed robustly in several recombinant expression systems used for the industrial production of commodity substances, such as enzymes used in the food industry (Zasloff, 2016). In those expression systems, the *Pichia pastoris* expression system with many advantages such as inexpensive culture to high cell densities, no toxicity from intracellularly accumulated materials, and easy purification has being used successfully for the production of various recombinant heterologous proteins (Damasceno et al., 2012). It is easily scaled-up to meet requirements of large-scale production. We have successfully expressed NZ2114 and MP1102 in *P. pastoris* during previous works (Zhang et al., 2014, 2015), especially NZ2114 achieved a high yield (1,309 mg/L). Hence, those AMPs could potentially be used by the dairy industry for they could be produced inexpensively and in large amounts.

## Conclusion

Based on all the evidence presented, results demonstrate that two plectasin-derived AMPs (NZ2114 and MP1102) had high efficacy *in vitro* (milk and bovine mammary epithelial cell) and *in vivo* (mouse model of mastitis) against *S. aureus*. These findings suggest that NZ2114 and MP1102 may be promising candidates for further development as a novel class of antimicrobials for intramammary therapy of *S. aureus*-induced mastitis.

## Author Contributions

LL, JW, and XZ designed the experiments, did the data analysis, and wrote the paper. LW and YG helped with the mouse mastitis experiment. All authors read and approved the final manuscript.

## Conflict of Interest Statement

The authors declare that the research was conducted in the absence of any commercial or financial relationships that could be construed as a potential conflict of interest.
